# Health behaviors and subsequent mental health problems during the COVID-19 pandemic: A longitudinal analysis of adults in the UK

**DOI:** 10.3389/fpubh.2022.1064677

**Published:** 2023-01-12

**Authors:** Kenisha Russell Jonsson, David C. Taylor-Robinson, Viviane Schultz Straatmann, Gabriella Melis, Nicholas Kofi Adjei

**Affiliations:** ^1^School of Public Health and Community Medicine, Institute of Medicine, University of Gothenburg, Göteborg, Sweden; ^2^Department of Public Health and Policy, University of Liverpool, Liverpool, United Kingdom; ^3^Department of Public Health Sciences, Stockholm University, Stockholm, Sweden; ^4^Leibniz Institute for Prevention Research and Epidemiology - BIPS, Bremen, Germany; ^5^Health Sciences Bremen, University of Bremen, Bremen, Germany

**Keywords:** COVID-19, health behaviors, mental health, physical activity, non-communicable diseases, alcohol, diet, pandemic mitigation

## Abstract

**Introduction:**

Public health mitigation policies aimed at slowing the spread of COVID-19 led to an increase in mental health problems (MHPs). This study examines the association between multiple pre-pandemic health behaviors and MHPs prior to, and during, the COVID-19 pandemic.

**Methods:**

We analyzed a representative population sample of 11,256 adults (aged 20–65 years) from Understanding Society—The UK Household Longitudinal Study. Baseline data from participants interviewed in 2017/2019 (wave 9) were linked to web surveys conducted during the COVID-19 pandemic. We used latent class analysis (LCA) to identify mutually exclusive health behavior (physical activity, alcohol consumption, eating habits and smoking tobacco) clusters by gender, and examined the sociodemographic correlates of each cluster. We assessed how pre-pandemic latent classes of health behaviors were associated with changes in MHPs during the pandemic using fixed effects regression models.

**Results:**

Three health behavior clusters were identified: positive (33%), moderate (24%), and high risk (43%), where similar behaviors clustered within individuals and sociodemographic circumstances. In particular, gender, age, migrant status and ethnicity were found to have strong associations with each cluster. Our results also demonstrated a clear association in MHPs with health behaviors both prior to, and during the pandemic. There were significant increases in MHPs between 2017/2019 and January 2021, with fluctuations coinciding with changes in public health mitigation policies. Assessments across the three clusters showed about 25.2%, 16.9%, and 0.7% increases in MHPs in the positive, moderate and high risk health behavior clusters, respectively.

**Discussion:**

This study shows that pre-pandemic health behaviors were significantly associated with mental health before and during the pandemic. Holistic policy interventions and promotions targeting multiple health behaviors may be an effective strategy to improve mental health in the pandemic recovery period.

## Introduction

Modifiable health behaviors such as physical inactivity, harmful consumption of alcohol, unhealthy dietary patterns and smoking tobacco increase the risk of non-communicable diseases (NCDs) (1, 2) including mental health problems (MHPs) (2–6), which are key contributors to death and disability in the UK and worldwide (1, 2, 7). There is good evidence linking health behaviors to MHPs, with studies demonstrating that unhealthy behaviors are associated with an increased risk of MHPs among adults (3–5, 8). Furthermore, prior studies demonstrate that NCDs including diabetes, stroke, cancer, and cardiovascular diseases, tend to co-occur, and share the same clustered risk factors often observed in people with poor mental health (1–8). Reducing exposure to unhealthy behaviors plays an integral role in the prevention and management of MHPs (3–6), attenuate mortality risks (9) and increase life expectancy for people with MPHs (10). A recent report from the World Health Organization (WHO) suggests that improving mental health is central to reducing the global burden of NCDs (2).

The COVID-19 pandemic has had ongoing impacts on both health behaviors and mental health (8, 11–19). Public health mitigation policies introduced during the course of pandemic included social distancing regulations, such as lockdowns restricting people's movement. These led to increased working from home, and the home schooling of children and young people became a part of daily life. Closure of health and sporting facilities, a reduction in time spent outdoors, and social distance and maximum occupancy rules were also part of the mitigation policies implemented during the pandemic. These mitigation measures resulted in substantial disruptions to daily life (18–21). Unintended consequences have included increased inequality in mental health and an increase in unhealthy behaviors (8, 22), both of which are socially patterned, with the greatest burdens experienced by those from lower socioeconomic backgrounds (11, 13–17). Evidence emerging from the pandemic indicates that health behaviors share several common determinants with mental health, such as education, employment, income, geography, gender and age (12, 23). It is therefore conceivable that changes in MHPs during the pandemic may have been shaped by the interaction between individual's health behaviors and their sociodemographic circumstances.

Existing studies assessing mental health and its association with health behaviors during the pandemic have been limited to analyses relying on cross-sectional data (22) or assessments of single/individual health behaviors (8, 14, 15, 17, 24). Yet, the literature demonstrates that health behavior patterns are distinct, and lie on a continuum between healthy and unhealthy (25–29). Moreover, the literature suggests that the co-occurrence of similar behaviors has a synergistic and multiplicative effect (25–30), which theoretically contributes to larger negative or positive health outcomes. Furthermore, previous studies have been limited to examining health behaviors concurrent with the pandemic rather than considering pre-pandemic health behaviors. Understanding the association between pre-pandemic health behaviors and MHPs may offer insights into how to implement policies and practices to support behaviors that may mitigate the short and long-term negative impacts of COVID-19 on mental health.

## Aim

This study aims to examine the association between multiple pre-pandemic health behaviors (physical activity, alcohol consumption, eating habits and smoking tobacco) and changes in MHPs during the first year of the COVID-19 pandemic (April 2020, May 2020, June 2020, July 2020, September 2020, November 2020, and January 2021) compared to pre-pandemic measurements collected in 2017–2019 (wave 9). We hypothesize that the pandemic may had a differential effect on MPHs for people based on their pre-pandemic health behaviors.

Two main research questions were addressed:

- How do pre-pandemic health behaviors cluster? And, to what extent does any clustering of health behaviors differ by gender, socio-economic and demographic factors?
- To what extent do MHPs prior to, and during the pandemic vary across any health behavior clusters identified?

**Figure 1:**
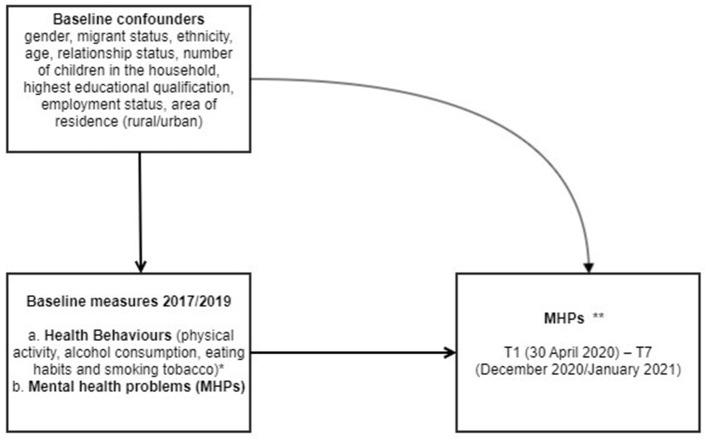
Directed acyclic graph for the current study. *Health behaviours assessed with 16 items capturing the frequency and/or intensity of the four behaviours mentioned. **Mental health Problems (MHPs) was measured at multiple time points throughout the pandemic: wave 1 (30 April 2020), wave 2 (May 2020), wave 3 (June 2020), wave 4 (July 2020), wave 5 (September 2020), wave 6 (November 2020) and wave 7 (December 2020/January 2021).

Figure 1 shows a directed acyclic graph which provides an overview of the outcome and exposures examined in the current study.

## Materials and methods

### Data and sample

We used data from Understanding Society—The UK Household Longitudinal Study, a nationally representative longitudinal household panel study based on a clustered-stratified probability sample of UK households, described in detail elsewhere (31). All adults (aged 16+ years) in chosen households were invited to participate. Data collection for each “wave” usually spans 24 months, with participants re-interviewed through online, face-to-face or telephone surveys. At the onset of the pandemic, additional data collections were performed to assess the impact of the pandemic on individuals, families and communities. Further details on data collection procedures, sampling and the indicators are detailed in the user guide (32). Pre-pandemic baseline data from 2017 to 2019 (wave 9), with household response rates of ~81%, were linked to seven waves of the COVID-19 survey. After linking the two data sets, only participants in both data files were kept (*n* = 17,946). However, participants 19 years or younger (*n* = 310), 66 years or older (*n* = 4,144), those missing responses on health behaviors (*n* = 40) and with missing weights (*n* = 3,453) were excluded from the analyses. The final dataset comprised 11,256 individuals (observations = 67,097).

#### Ethical approval

The University of Essex Ethics Committee approved all data collection for the Understanding Society main study and the COVID-19 surveys (32). No additional ethical approval was necessary for this secondary data analysis.

### Measures

#### Mental health problems (MHPs)

Self-reported mental health was measured using the General Health Questionnaire-12 (GHQ-12) available in wave 9 and the COVID-19 surveys. GHQ-12 is a unidimensional measure of general mental health (33), and includes items relating to: problems enjoying day-to-day activities; not feeling useful; problems concentrating; general happiness; sleeplessness, feeling unhappy or depressed; inability to decide; inability to face problems; feeling constantly under strain; feeling worthless; losing confidence; and problems overcoming difficulties. MHP was assessed using the sum of the GHQ-12 score as a continuous measure with items rescaled to a range from 0 to 100, with higher values indicating higher level of MHPs and thus poor mental health.

#### Health behaviors

Four health behaviors (physical activity, alcohol consumption, eating habits and smoking tobacco) comprising 16 items were analyzed. Three physical activity categories were created from the short form of the International physical activity questionnaire (IPAQ) (33), which asked respondents to account for the number of days they walked, and/or engaged in moderate or vigorous physical activities during the last 7 days for at least 10 min at a time. Based on the intensity and duration of the respondents' level of physical activity, they were categorized as engaging in low, moderate or high levels of physical activity. Alcohol consumption was measured using an adapted self-reported version of the Alcohol Use Disorders Identification Test for Consumption (AUDIT-C) instrument (34). Respondents were asked about the frequency and quantity of alcohol they consumed in the past 12 months, and their responses were categorized as low, moderate or high risk drinkers with possible alcohol dependency. Eating habits was assessed by two separate indicators on the frequency of fruit and vegetable consumption during a usual week, with responses coded as none, 1–3 days per week, 4–6 days per week, or every day. The first two categories were combined into a new category for all respondents who consumed fruit/vegetables 0–3 days per week because few respondents reported not eating any fruit or vegetables (3, 35–38). Smoking tobacco was based on whether respondents smoked cigarettes (not including e-cigarettes) and the number of cigarettes they usually smoked in a day. These responses were coded as none/light smokers (smoking between 0 and 10 cigarettes per day), average smokers (11–19 cigarettes per day) and heavy smokers (20 or more cigarettes per day) (30).

#### Sociodemographic indicators

Sociodemographic indicators identified in earlier studies as being associated with health behaviors and/or mental health were included in the analysis (28, 29). These were gender, migrant status, ethnicity, age, age-squared, relationship status, number of children in the household, socioeconomic status (measured by highest educational qualification, employment status), and area of residence (rural/urban). For sub-group analyses, we used an additional measure examining vulnerability to COVID-19 with individuals categorized as having low/normal or high risk. In the UK, individuals with long term health and rare conditions, such as cystic fibrosis, sickle cell or H.I.V. were categorized as having a high risk for poor COVID outcomes. Further details on this classification may be found in the Understanding Society COVID-19 guide (32).

### Analytical strategy

Statistical analyses were conducted in 3 stages. First, a series of Latent class analyses (LCA) were used to identify homogenous and mutually exclusive but distinct health behavior clusters, for the total sample and by gender. The clusters were based on the underlying composite patterning of four health behaviors (physical activity, alcohol consumption, eating habits and smoking tobacco) from a total of 16 items. All LCA models were estimated using PROC LCA Version 1.2.5, a SAS procedure for conducting LCA (39). Models were estimated using an expectation–maximization (EM) algorithm with 1,000 starts, and model adjustments for unequal selection probabilities, differential non-response and the complex survey design made by incorporating cross-sectional survey weights from Wave 9 of the main survey (32, 40). A series of models were estimated until the inclusion of an additional latent cluster did not lead to significant improvements in model fit (41). Model fit was determined with the combination of the G-square (G^2)^ statistic and the following information criteria: Akaike information criterion (AIC), Bayesian information criterion (BIC), Consistent Akaike information criterion CAIC, and the Adjusted bayesian information criterion (aBIC). Final model selection was also based on interpretability; that is, whether the latent clusters showed distinct and logical patterns that could readily be described.

In the second stage of the analysis, logistic regression was used to examine the sociodemographic correlates of each identified cluster. In these models, we assessed the odds of cluster membership with 95% confidence intervals (CI) based on sociodemographic exposures.

In the third and final stage of the analysis, weighted fixed effects regression models were applied to assess the relationship between our identified LCA behavior clusters and changes in MHPs (outcome) during the first year of the pandemic relative to pre-pandemic MHPs. The models assessed the increase or decrease in MHPs during the time periods under comparison over the period of the pandemic, and whether these differences vary across each of the identified pre-pandemic health behavior clusters. Age and age-squared were included in all models to control for normal fluctuations in mental health due to aging (42). Analyses were performed using STATA version 16 (43).

## Results

### Descriptive characteristics of the analytical sample at baseline

Table 1 shows the descriptive characteristics of the total analytical sample and the three identified health behavior clusters. The sample consisted of 51.7% females, 12.0% from ethnic groups other than white, 11.4% non-UK born migrants. 42.5% of individuals in sample were single/never married and 46.6% were married/cohabiting whilst 10.9% were divorced/separated or widowed. 67.6% of the sample had no children while 32.4% had at least one child. The overall age distribution was fairly equal with ~20% of individuals in each 10 year age group from age 20–49. However, 25% of the sample were between the ages 50–59 and 13% were between ages 60–65. 37.4% had obtained some form of college level qualifications (A-level equivalent) while 29.6% had degree level education or higher, 28.1% had O-level education and 4.8% had some other form of qualification or none at all. The majority of respondents were economically active (self-employed 8.7% or in paid employment 65.6%) whilst 25.7% were not in the labor market. The distribution of the sample across the three emergent clusters showed some variation. More men, people born in the UK, individuals aged 20–29 and 30–39, single/unmarried people and those having no children were categorized as being in cluster 3 (*high risk health behaviors*).

**Table 1:** Baseline characteristics of total sample and across the identified latent health behavioral clusters (cross-sectional weights).

	**Total sample**		**Cluster 1**		**Cluster 2**		**Cluster 3**	
	***n***	**%**	***n***	**%**	***n***	**%**	***n***	**%**
**Gender**								
Men	5,433	48.3	1,407	48.9	1,733	40.4	2,204	53.9
Women	5,823	51.7	1,473	51.1	2,557	59.6	1,882	46.1
**Migrant status**								
UK born	9,814	88.6	2,523	88.7	3,601	85.3	3,653	91.2
Not UK born	1,259	11.4	322	11.3	620	14.7	354	8.8
**Ethnicity**								
White	9,813	88.0	2,542	88.9	3,729	87.7	3,544	87.8
Non-whites	1,338	12.0	318	11.1	524	12.3	494	12.2
**Age groups**								
20–29	2,450	21.8	598	20.8	630	14.7	1,133	27.7
30–39	2,111	18.8	554	19.2	669	15.6	853	20.9
40–49	2,392	21.3	606	21.0	981	22.9	823	20.1
50–59	2,804	24.9	780	27.1	1,253	29.2	835	20.4
60–65	1,499	13.3	342	11.9	756	17.6	443	10.8
**Relationship status**								
Single/never married	4,767	42.5	1,179	41.1	1,407	32.9	2,060	50.6
Married/cohabit	5,228	46.6	1,422	49.6	2,391	55.8	1,541	37.9
Divorced/separated/widow	1,223	10.9	266	9.3	484	11.3	468	11.5
**Number of children**								
0	7,606	67.6	1,901	66.0	2,873	67.0	2,815	68.9
1	1,497	13.3	389	13.5	570	13.3	538	13.2
2	1,601	14.2	439	15.2	666	15.5	517	12.7
3 or more	553	4.9	151	5.3	180	4.2	215	5.3
**Highest educational qualification**								
Degree or higher (or equivalent)	3,298	29.6	935	32.7	1,676	39.5	821	20.3
Higher education or A level equivalent	4,172	37.4	1,097	38.4	1,433	33.8	1,606	39.7
O-level or equivalent	3,134	28.1	707	24.8	981	23.1	1,368	33.8
Other or none	539	4.8	118	4.1	151	3.6	251	6.2
**Employment status**								
Self employed	979	8.7	235	8.2	447	10.4	314	7.7
Paid employment	7,383	65.6	2,025	70.4	2,888	67.4	2,519	61.7
Unemployed	573	5.1	114	4.0	145	3.4	287	7.0
Economically inactive	1,523	13.5	302	10.5	541	12.6	650	15.9
Student training or doing something else	790	7.0	202	7.0	265	6.2	313	7.7

### Distribution of the item-response probabilities and class-membership

**Figure 2:**
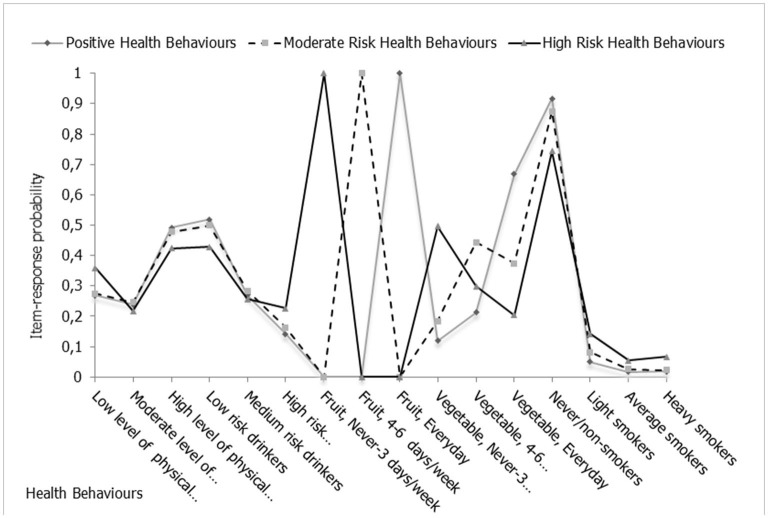
Item-response probabilities for three-cluster model. Probability of reporting certain health behaviors given latent cluster.

Figure 2 presents an overview of the item-response probability for the 16 health behavior items. This is the probability that respondents indicated that they engaged in particular health behaviors, that is, “yes” responses across the three identified latent clusters. Assessing responses related to physical activity, it appears that the majority of participants engaged in high levels of physical activity, with fewer respondents engaging in low to moderate levels of physical activity. In *cluster 1* and *cluster 2*, ~50% of individuals engaged in high levels of physical activity (with 48 and 49% responding “yes”). As it relates to alcohol consumption, the majority of low risk drinkers were in *cluster 2* (52%) while the majority of moderate (28%) and high risk drinkers with possible dependency (23%) were found in *cluster 1* and *cluster 3*, respectively. Responses in relation to the consumption of fruits and vegetables were the most distinctive items across the clusters. For instance, the majority of individuals in *cluster 2* responded that they ate fruits (100%) and vegetables (67%) every day. In contrast, respondents in *cluster 1* indicated a more moderate consumption of fruits (100%) and vegetables (44%) between 4 and 6 days per week. There was a high probability that the respondents in *cluster 3* never and/or consumed fruits (100%) and vegetables (50%) 3 days per week or less. The patterns related to smoking tobacco was found to be similar to the other items described, where individuals classified in *cluster 2* were predominantly never-smokers/non-smokers, individuals in *cluster 1* were light respective average smokers whilst respondents in *cluster 3* had a higher probability of being average to heavy smokers.

It appears that *cluster 2* consisted mainly of individuals engaging in moderate and/or high levels of physical activity, minimum alcohol consumption, healthy eating habits and non-smoking. Individuals categorized in *cluster 1*, engaged in a mix of healthy behaviors including the moderate consumption of alcohol, they were light smokers, but, they also engaged in unhealthy behaviors such as moderate levels of physical activity and they consumed fruits and vegetables ~4–6 days per week. *Cluster 1* engagement in healthy behaviors were at a lower frequency and intensity than individuals in the *cluster 2*. In contrast, *cluster 3* consisted of individuals that were physically inactive, had high alcohol consumption and/or dependency, engaged in poor eating habits and were smokers.

The results indicate that there are key differences in the clustering of health behaviors across the total sample. This difference is exemplified in the consumption of fruits. Individuals in each of the clusters were found to consume fruits daily, frequently or infrequently/not at all. The results also indicated that the item-response probabilities varied by gender (Supplementary Figures S1, S2). Approximately 13% more men reported having high levels of physical activity than women, meanwhile, women were more likely to be low risk drinkers. There was 11 percentage point difference in the probability of consuming alcohol when women and men were compared. The consumption of fruits was found to be one of the strongest differences in the health behaviors of men and women. Women were more likely to consume fruits 4–6 days per week (37.3 vs. 32%) or everyday (61.6 vs. 48%) compared to men.

**Figure 3:**
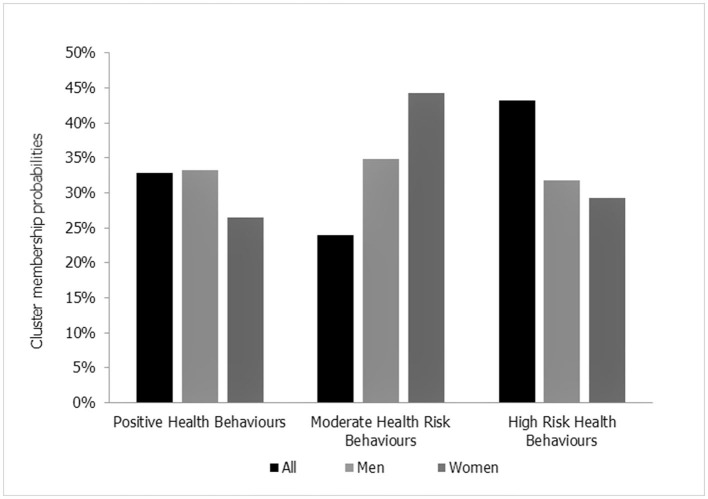
Cluster membership probabilities for the total population and by gender.

Based on the item-response probabilities and the fit indices (Supplementary Table S2), a model with three clusters had the best fit. Figure 3 shows the distribution of the three identified latent clusters for the total sample and by gender. The clusters were named according to the most prominent health behavioral characteristics:

*Cluster 1*—the “*Moderate risk health behaviors*,” comprised 24% of the total sample. Classification by gender indicated that this cluster consisted of 35% men and 44% women.
*Cluster 2*—the “*Positive health behaviors*,” comprised 33% of the total sample. Classification by gender indicated that this cluster consisted of 33% men and 26% women.
*Cluster 3*—the “*High risk health behaviors*,” comprised 43% of the total sample. Classification by gender indicated that this cluster consisted of 32% men and 29% women.

The differences in item-response probabilities in health behaviors among men and women meant that 7% less women than men were classified as engaging in positive health behaviors whilst 9% more women than men were classified as having moderate risk health behaviors.

### Sociodemographic association with cluster membership

The full results of the association between the sociodemographic indicators and cluster membership are shown in Table 2. Overall, the results demonstrated strong associations between cluster membership and gender, ethnicity, migrant status, relationship status, geography, number of children and age. However, these results varied across the identified clusters. In particular, we found that individuals in the *positive health behavior cluster* had the highest odds of being women, non-UK born migrants, older people aged between 50 and 65 years, White, married or in a cohabiting partnership, to have at least two children, residence in rural areas, to have at least a degree level education or to be self-employed, when compared to the other two clusters. Individuals in the *moderate risk health behavior cluster* were more likely to be men, individuals born in the UK, in paid employment or student training, and people aged between 30 and 59 years old. Strong associations were found between classification in the *high risk health behavior cluster* and the likelihood that the individual was male, born in the UK, self-identifying as non-White, between the ages of 20–39, unemployed, and having a high vulnerability to COVID-19. The relationship between health behaviors and having children was somewhat mixed, but it appears that individuals with two children were the least likely to engage in *high risk health behaviors*.

**Table 2:** Sociodemographic indicators and latent clusters membership.

	**Positive health behaviors**			**Moderate health risk behaviors**			**High risk health behaviors**		
	**Odds ratio**	**95%**	**CI**	**Odds ratio**	**95%**	**CI**	**Odds ratio**	**95%**	**CI**
**Gender**									
Men	0.60	0.55	0.65	1.12	1.03	1.22	1.52	1.41	1.65
Women	1.67	1.55	1.81	0.89	0.82	0.97	0.66	0.61	0.71
**Migrant status**									
UK born	0.76	0.68	0.85	1.12	0.98	1.27	1.23	1.09	1.38
Not UK born	1.32	1.18	1.48	0.90	0.79	1.02	0.81	0.72	0.92
**Age groups**									
20–29	0.50	0.44	0.57	0.96	0.84	1.09	1.91	1.71	2.14
30–39	0.74	0.67	0.82	1.05	0.94	1.17	1.29	1.17	1.42
40–49	1.00	0.91	1.09	1.06	0.96	1.17	0.95	0.87	1.04
50–59	1.25	1.15	1.35	1.08	0.99	1.19	0.74	0.68	0.81
60–65	1.59	1.44	1.76	0.80	0.71	0.90	0.73	0.65	0.81
**Ethnicity**									
White	1.20	1.07	1.33	1.06	0.94	1.20	0.80	0.72	0.88
Non-whites	0.84	0.75	0.93	0.94	0.83	1.06	1.26	1.13	1.40
**Relationship status**									
Single/never married	0.63	0.57	0.68	1.00	0.92	1.10	1.57	1.45	1.71
Married/cohabit	1.49	1.38	1.61	1.05	0.97	1.15	0.64	0.59	0.69
Divorced/separated/widow	0.99	0.88	1.11	0.88	0.77	1.00	1.12	1.00	1.26
**Number of children in the household**									
0	0.97	0.90	1.05	0.96	0.88	1.05	1.06	0.98	1.15
1	0.92	0.82	1.02	1.03	0.92	1.17	1.06	0.95	1.18
2	1.14	1.02	1.26	1.08	0.96	1.21	0.82	0.74	0.91
3 or more	0.98	0.82	1.17	0.86	0.70	1.06	1.15	0.96	1.37
**Rural/Urban**									
Rural	1.37	1.25	1.49	0.92	0.83	1.02	0.77	0.70	0.84
Urban	0.73	0.67	0.80	1.08	0.98	1.20	1.30	1.19	1.43
**Highest educational qualification**									
Degree or higher (or equivalent)	1.73	1.60	1.87	1.11	1.02	1.21	0.51	0.47	0.55
Higher education or A level equivalent	0.77	0.71	0.83	1.01	0.93	1.11	1.29	1.20	1.40
O-level or equivalent	0.70	0.64	0.77	0.89	0.80	0.99	1.55	1.41	1.69
Other or none	0.69	0.53	0.89	0.69	0.51	0.93	1.85	1.45	2.35
**Employment status**									
Self employed	1.25	1.10	1.42	0.88	0.76	1.01	0.88	0.77	1.00
Paid employment	1.00	0.92	1.08	1.27	1.16	1.39	0.83	0.76	0.90
Unemployed	0.60	0.47	0.76	0.84	0.65	1.08	1.81	1.47	2.23
Economically inactive	1.16	1.04	1.29	0.70	0.62	0.80	1.13	1.01	1.26
Student training or doing something else	0.63	0.52	0.75	1.08	0.90	1.30	1.44	1.22	1.70
**Vulnerability to COVID**									
Low/normal risk	0.97	0.82	1.15	1.37	1.12	1.67	0.81	0.69	0.96
High risk	1.03	0.87	1.21	0.73	0.60	0.90	1.23	1.04	1.45

### Changes in MHPs across health behavior clusters

**Figure 4:**
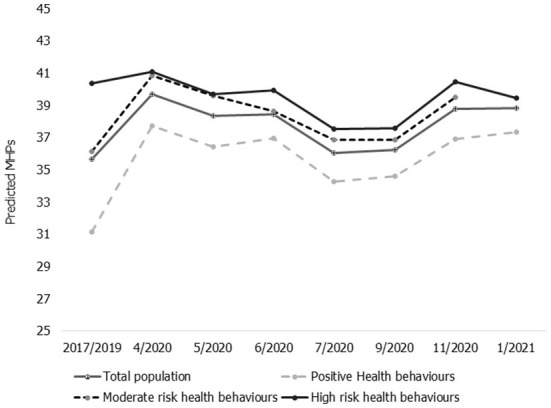
Changes in MHPs during the COVID-19 pandemic by health behavior clusters. MHPs (scale 0–100) during the first year of the COVID-19 pandemic (4/2020–01/2021) in the UK. Relative to pre-pandemic measures 2017/2019 (fixed effects regression of the same person, adjusted for general changes in age, age-squared and weighted with each person's last individual weight). Total population = the total sample.

Figure 4 shows the average within person changes in MHPs across the general population and for the three health behavior clusters. Assessments across the three clusters showed about 25.2%, 16.9%, and 0.7% increases in MHPs in the positive, moderate and high risk health behavior clusters, respectively.

Prior to the pandemic, individuals classified as having *positive health behaviors* had lower average MHPs compared to the general population (29.7 points vs. 33.3 points, out of a possible of 100) at baseline measured in 2017/2019. However, MHPs in this cluster increased by ~5.6 points during the first 3 months of the COVID-19 pandemic. Although there were some improvements in mental health for this cluster between July and September of 2020, this was short lived, with significant increases in MHPs to 37.2 points as measured in January 2021.

Compared to the other health behavior clusters, people in the *positive health behaviors* cluster had the largest increase in MHPs during the pandemic. Pre-pandemic MHPs in this group were 5.3 and 7.6 points lower when compared to the *moderate* and *high risk health behaviour clusters*, respectively. This difference reduced to less than a 1 percentage point difference in January 2021. MHPs increased steadily in the *moderate risk health behaviors cluster* with the largest increase at the start of the pandemic. Compared to pre-pandemic assessments MHPs in the *moderate risk health behaviors cluster* increased from 32.0 points to 36.9 points, a difference of 4.9 points, and was 37.2 points in January 2021. In contrast to the other clusters, MHPs remained consistently high among individuals classified as having *high risk health behaviors*. In addition, this cluster had the smallest changes in MHPs from pre-pandemic levels and throughout the pandemic. Pre-pandemic MHPs within the *high risk cluster* (37.3 points) were 4.0 points higher than the population average, and at 37.6 points at the last measurement in January of 2021.

**Figure 5:**
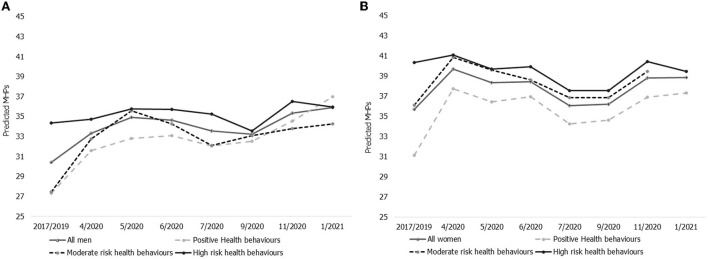
**(A)** Changes in MHPs during the COVID-19 pandemic by health behavior clusters among men. MHPs (scale 0–100) during the first year of the COVID-19 pandemic (4/2020–01/2021) in the UK. Relative to pre-pandemic measures 2017/2019 (fixed effects regression of the same person, adjusted for general changes in age, age-squared and weighted with each person's last individual weight). **(B)** Changes in MHPs during the COVID-19 pandemic by health behavior clusters among women. MHPs (scale 0–100) during the first year of the COVID-19 pandemic (4/2020–01/2021) in the UK. Relative to pre-pandemic measures 2017/2019 (fixed effects regression of the same person, adjusted for general changes in age, age-squared and weighted with each person's last individual weight).

The impacts of the pandemic on MHPs across the cluster for men and women are shown in Figures 5A, B. These results indicate that the pattern of changes in MHPs for both men and women was similar to the overall within and between cluster changes in MHPs during the pandemic. However, pre-pandemic levels of MHPs were higher for women across all the clusters when compared to men. In particular, we observed that women in the *moderate risk health behavior cluster* had 8.7 points higher scores than the pre-pandemic levels of men (36.1 points among women vs. 27.5 points among men). This difference reduced gradually during the pandemic from May 2020 (4 points difference) but the gap began to increase again in November 2020. There was a similarly large gap in MHPs between men and women in the *high risk health behavior cluster*, and this also reduced gradually *during the* pandemic from pre-pandemic levels in 2017/2019 to 3.5 points in January 2021. The gap in MHPs by gender was smallest in the *positive health behavior* cluster. Assessments of the percentage change in MHPs measured in 2017/2019 compared to January 2021, across the three clusters and among men, showed about 35.4, 24.7, and 4.7% increases in MHPs in the positive, moderate and high risk health behavior clusters, respectively. Among women, MHPs increased by 19.9 and 12.3% in the positive and moderate risk health behavior clusters (respectively) but decreased (−2.3%) in the high risk health behavior cluster.

## Discussion

This study examined the co-occurrence, clustering and social patterning of multiple pre-pandemic health behaviors and subsequent MHPs during the COVID-19 pandemic. Using a nationally representative study, Understanding Society—The UK Household Longitudinal Study, we identified three distinct health behavior clusters: positive (33%), moderate (24%) and high risk (43%), and assessments across these clusters indicated 25.2%, 16.9%, and 0.7% increases in MHPs in the positive, moderate and high risk health behavior clusters, respectively. Our results further showed a clear association in MHPs with health behaviors both prior to, and during the pandemic with significant increases in MHPs between 2017/2019 and January 2021.

Consistent with the results of previous studies (22–26), we found that similar health behaviors clustered and co-occurred within individuals. This is despite the differences in research methodology (settings, analytical tools, data source) and operationalization of health behaviors in this study compared to earlier studies. These findings show that individuals may be categorized according to specific behavioral typologies when similar unobserved patterns of behaviors among individuals are identified. Our results also suggest significant associations between cluster membership and a variety of individual sociodemographic indicators. For instance, individuals in the *high risk health behavior* cluster were likely to be men, migrants, young, divorced/single and to be economically inactive or students. These results corroborate studies showing that the clustering of health behaviors is not random, and that there is a greater prevalence/stronger association among people sharing similar sociodemographic circumstances (25, 27, 29).

We found that 43% of the total sample were likely to engage in *high risk health behaviors*. This is the unhealthiest group, comprising the highest proportion of physically inactive individuals, high risk drinkers, smokers and those with poor eating habits. The healthiest third of the total sample (33%), labeled the *positive health behaviors cluster*, consisted of individuals engaging in high levels of physical activity, low risk drinking, high daily intake of fruits /vegetables and low likelihood of tobacco smoking. Our results show that ~24% of the total sample were engaged in *moderate health behaviors*. Individuals in this cluster engaged in a mix of unhealthy behaviors (that is, they consumed alcohol and smoked tobacco) but they also engaged in healthy behaviors such as moderate levels of physical activity and consumed fruits and vegetables 4–6 days per week on average.

Our findings also point to an important gender dimension to the clustering of health behaviors. This is finding is consistent with previous studies which highlight the gender gap in health behaviors (26–29, 44). Women were more likely to be categorized as engaging in the *moderate* (44 vs. 35%) and positive (26 vs. 33%) health behavior clusters when compared to men. This suggests that compared to women, men were more equally distributed across the three clusters. Comparisons across individual health behaviors indicated that men were more likely to engage in intense physical activity but were less likely to consume fruits and vegetables on a daily basis. Graham et al. (44) identified similar behaviors in their study. Prior studies also pointed to strong patterns of co-occurrence between certain health behaviors, for instance, alcohol misuse and smoking; or having an unhealthy diet and smoking (26, 29, 44), especially among women. These relationships were however not evident in this study. A likely explanation may be the fact that smoking was not a particularly strong indicator of cluster membership.

During the three key lockdown periods, March 2020, November 2020 and January 2021, the government introduced a number of public health mitigation measures. These included mandating that people work from home, and the closure of schools, care centers, and non-essential services (e.g., clothing stores, gyms). Furthermore, regulations during the height of the pandemic included social distance and maximum occupancy rules and people were advised to limit contact with those outside their household. The pandemic therefore led to massive disruptions to all aspects of daily life, and this contributed to changes in health behaviors, as well as contributing to significant increases in poor mental health. Baseline assessment of the relationship between MHPs and our emergent health behavior clusters found strong associations. Engaging in positive health behaviors was associated with the lowest level of MHPs, while individuals engaged in high risk health behaviors were found to have highest MHPs, and thus poorer mental health. In comparison to the baseline assessments, our results indicated that pre-pandemic engagement in *positive health behaviors* was linked with the steepest increase in MHPs during the pandemic, followed by individuals classified as having *moderate risk health behaviors*. The pandemic appeared to have had a smaller negative impact on MHPs among individuals from the *high risk health behavior* cluster. Overall, the pre-pandemic differences in mental health observed between the clusters was greatly reduced during the pandemic.

Additional models examining probable gender differences across the health behavior clusters indicated that the steepest increase in MHPs was for women. Similar gender differences have been found in other studies (12, 16). In addition to the wider societal changes described above, there is literature showing that the pandemic and the public mitigation measures had different impacts on the mental health of men and women. Already at the start of the pandemic women were shown to suffer from more anxiety (20), but, the worsening mental health may partly be explained by women's roles both in the labor market and in the household. They represented 58% of all key/frontline workers and were most represented in education and childcare (81%), and health and social care (79%) (21). Simultaneously, women were more likely to work in sectors affected by job losses due to shutdowns (17% women compared to 13% men) (18). At the household level, among parents, school and childcare closures meant that children required an additional 6 h of care (18, 19). The added stress of taking on multiple roles simultaneously may have been a contributing factor in the increased poor mental health among women. The results demonstrate that the effects of the pandemic were not equal, because some groups experienced a disproportionate decrease in their mental health, corroborating findings from earlier studies (12, 13, 16).

Taken together, the results suggest that individuals who had the opportunity to, and who normally engaged in healthy behaviors may have adjusted their behaviors because of the pandemic and the public health mitigation policies (8, 12, 14, 41), and contributed to increasingly poor mental health (11, 13, 15, 17). It is therefore likely that health promoting policies that increase opportunities for people with unhealthy behaviors to engage in healthy behaviors could substantially reduce health inequalities both in short and long-term. Although, we did not explicitly test for changes in health behaviors during the pandemic, changes in mental health over the period of the pandemic may partly be explained by changes in health behaviors (8, 14, 17, 24). There were notable fluctuations in mental health throughout the pandemic, and these coincided with public health mitigation measures. The most significant increases in MHPs coincided with increasingly restrictive mitigation regulations while there were noticeable improvements when restrictions were lifted. This is evidenced by a reduction in MHPs when the first wave of the pandemic subsided (July/September 2020) and some of the restrictions were lifted. However, coinciding with the second national lockdown in November 2020, MHPs increased once more. Similar periods of fluctuations in mental health (14) and health behaviors (24) were highlighted in other studies. It should however be noted, that good levels of mental health did not return to pre-pandemic levels at the last available data point in this study. The question, therefore is whether the effects of the pandemic on mental health are short-term and whether people will return to pre-pandemic levels when restrictions are completely lifted, and society returns to “normal.”

### Study strengths and limitations

One of the limitations of the study relates to low survey response rates over the pandemic period. The 2017–2019 (wave 9) survey had a response rate of ~84%, compared to a response rate of 35% at the first wave of the COVID-19 survey. Response rates may reflect an under-representation of individuals who were ill due to COVID-19, other pandemic effects may include the significant changes in the home life of respondents, for e.g., parents of young children were much busier during lockdowns when schools and childcare centers were closed. Nonetheless, population non-response weights were included in the analyses, and as such the study should generally be representative of the UK population. Moreover, because the data is longitudinal, it was possible to examine the mental health of survey participants before the pandemic and not simply assess mental health concurrent with the pandemic.

There were a number of limitations related to the measures used in the study. There may be recall bias from related to GHQ-12 and health behaviors. In addition, there are a number of health behaviors shown to be associated with mental health, for example sleep and sedentary behaviors, which have not been included in this study due to a lack of available data in the waves examined. Moreover, health behaviors were not measured during the pandemic, as such, it was not possible to look at changes in health behaviors over time or their relationship with MHP.

Another limitation is that there are no perfect fit indices when assessing the latent clusters. However, using multiple indices allowed for a more balanced assessment of the LCA models. BIC is an appropriate fit index because it penalizes model complexity to avoid overfitting in large samples (41, 45, 46). In contrast, AIC, a commonly used fit criterion has been suggested to over fit larger samples (41, 46). As it relates to the models, fixed effects models do not take into account changes in health behaviors that may have occurred during the pandemic. There are studies indicating that public health mitigation measures have led to changes in health behaviors (12, 14, 15, 17). Our fixed effects models have a key strength in controlling for potential bias due to time invariant unobserved heterogeneity (for e.g., personal traits, childhood life circumstances) and omitted variable bias (47). At the same time, it is not possible to fully exclude unobserved predictors or missing data.

An important consideration to bear in mind, is that the potential margin of change in mental health was not the same for all the clusters. It is likely that individuals classified in the *high risk health behaviors cluster* already faced major social, economic and even mental health challenges prior to the pandemic. The low levels of change suggest a certain level of stability in the reporting of MHPs. The observed changes in the *positive* and *moderate* health behavior clusters, may also be interpreted as an indication of individuals who were potentially vulnerable to developing poor mental health.

Despite the drawbacks, a key strength of the current study includes the use of a large and nationally representative sample. To our knowledge, this study is the first to assess the relationship between multiple pre-pandemic health behavior clusters and changes in MHPs over the pandemic period. By exploring the association between the health behavior clusters and sociodemographic characteristics, it was possible to identify the ways in which health behavior patterns differed by social circumstances. In addition, the data is longitudinal, covering approximately a year of the pandemic and one pre-pandemic measurement. Using this data permitted analyses of within-person changes, which provides a more accurate indication than is otherwise currently available of pre-pandemic health behaviors and its association with MHPs during the pandemic. Analysis of the data using a LCA adds to the growing scientific literature suggesting that health behaviors are not randomly distributed, rather they cluster and co-occur within individuals and sociodemographic groups (25–29). An advantage of using LCA is the possibility to examine the inter-linking relationships between multiple health behaviors simultaneously. With this method, it is possible to assess multiple combinations of unobserved patterns, before reducing the data into smaller behavioral clusters (45, 48).

## Conclusion

The results demonstrate that using person-centered methods in the assessment of multiple health behaviors may provide a guide to prioritizing the components and/or types of policy interventions required to improve and foster healthy behaviors. Moreover, assessments of the clustering of health behaviors offer insights on the potential cumulative impact of multiple interacting factors and whether these have a greater detrimental or beneficial impact on health when compared to single/individual health behaviors. Our study results further demonstrated clear differences in pre-pandemic MHPs on the basis of health behaviors and individual social circumstances. These differences remained even with the notable increases in poor mental health during the pandemic.

Our findings show short and long-term negative mental health consequences even after the end of the pandemic. Individuals from the *high risk health behavior* cluster represents an important target group for policy interventions given that individuals with unhealthy behavioral practices are overrepresented across a wide number of NCD related illnesses. Creating effective policies to address health behavior change is also relevant for individuals from the *moderate risk health behavior* cluster. Although they require less intensive interventions, individuals from this cluster may benefit from health promoting interventions aimed at increasing the frequency and intensity of their engagement in healthy behaviors. Engaging in positive *health behaviors* 2 years prior to the pandemic is associated with better overall mental health prior to, and during the pandemic. This suggests that despite the sharp increase in poor mental health in this cluster during the pandemic, engagement in healthy behaviors may have a mitigating impact. From a policy perspective, our results indicate that holistic policy interventions and interventions targeting multiple health behaviors are important components for improving the post-COVID population mental health.

## Data availability statement

The data analyzed in this study is subject to the following licenses/restrictions: Understanding Society deidentified survey participant data (URLs: http://doi.org/10.5255/UKDA-SN-6614-13; http://doi.org/10.5255/UKDA-SN-8644-3) are available from the UK Data Service. Researchers are required to register with the UK Data Service and complete the relevant information before being allowed to download the datasets. Requests to access these datasets should be directed to (URL: https://ukdataservice.ac.uk/).

## Ethics statement

Ethical review and approval was not required for the study on human participants in accordance with the local legislation and institutional requirements. The patients/participants provided their written informed consent to participate in this study.

## Author contributions

KR conceived the idea for the study, conducted the analyses, and drafted the manuscript. KR and NA contributed to the interpretation of the findings. KR, NA, DT-R, VS, and GM critically revised the manuscript. All authors approved the final version of the paper.
