# Supplementary material for: Health behaviors and subsequent mental health problems during the COVID-19 pandemic: A longitudinal analysis of adults in the UK
Source: Front Public Health. 2023 Jan 12;10:1064677. doi: 10.3389/fpubh.2022.1064677 (PMC9877513; doi:10.3389/fpubh.2022.1064677)
Supplement: Supplementary file 1 [file Data_Sheet_1.pdf]

Supplementary appendix: Health behaviours and subsequent mental health problems during the COVID-19 pandemic: A longitudinal analysis of adults in the UK

Supplementary Table 1. Characteristics of total sample across the health behavioural classes. (Longitudinal weights)

	Total Sample		Positive Health Behaviours		Moderate Risk Health Behaviours		High Risk Health Behaviours	
	n	%	n	%	n	%	n	%
**Gender**								
Men	22532	47.0	7474	38.2	6133	49.6	6133	49.6
Women	25379	53.0	12100	61.8	6241	50.4	6241	50.4
**Migrant Status**								
UK born	43374	91.8	17402	90.1	11197	91.6	11197	91.6
Not UK born	3888	8.2	1921	9.9	1033	8.4	1033	8.4
**Age groups**								
20-29	10131	21.40	3080	15.94	2704	22.09	2704	22.09
30-39	9204	19.45	3239	16.77	2414	19.72	2414	19.72
40-49	10318	21.80	4050	20.96	2584	21.11	2584	21.11
50-59	11944	25.23	5855	30.31	3239	26.46	3239	26.46
60-65	5735	12.12	3094	16.01	1302	10.63	1302	10.63
**Ethnicity**								
White	43218	90.7	17940	92.1	11280	91.7	11280	91.7
Non-whites	4414	9.3	1530	7.9	1025	8.3	1025	8.3
**Relationship status**								
single/never married	20497	42.9	6614	33.8	5308	43.1	5308	43.1
maried/cohabit	22145	46.4	10688	54.7	5889	47.8	5889	47.8
divorced/sparated/widow	5118	10.7	2247	11.5	1111	9.0	1111	9.0
**Number of children in the household**								
0	33680	70.3	13964	71.4	8852	71.6	8852	71.6
1	5635	11.8	1919	9.8	1410	11.4	1410	11.4
2	6698	14.0	2942	15.0	1705	13.8	1705	13.8
3 or more	1879	3.9	741	3.8	402	3.3	402	3.3
**Higest educational qualification**								
Degree or higher (or equivalent)	15195	31.9	8167	42.0	4236	34.4	4236	34.4
Higher education or A level equivalent	18311	38.5	6836	35.1	5059	41.1	5059	41.1
O-level or equivalent	12784	26.9	4166	21.4	2730	22.2	2730	22.2
Other or none	1311	2.8	282	1.4	280	2.3	280	2.3
**Employment status**								
Self employed	3907	8.2	1806	9.2	925	7.5	925	7.5
Paid employment	32346	67.6	13250	67.7	9160	74.0	9160	74.0
Unemployed	2139	4.5	543	2.8	430	3.5	430	3.5
Economically inactive	5928	12.4	2613	13.4	1028	8.3	1028	8.3
Student.training or doing something else	3557	7.4	1346	6.9	831	6.7	831	6.7

Source: Authors' analysis Understanding Society mainstage survey in 2017/2019 and Understanding Society: COVID-19 Study, waves 1-7

Supplenentary Table 2. Indicators of fit for models with one through five latent classes

Panel A. Fit indices for the Total Sample						
**Number of classes**	**G-squared**	**AIC**	**BIC**	**CAIC**	**aBIC**	***df***
**1**	82632.0	82664.0	82781.2	82797.2	82730.4	65519
**2**	69120.4	69186.4	69428.2	69461.2	69323.4	65502
**3**	59357.5	59457.5	59824.0	59874.0	59665.1	65485
**4**	54898.8	55032.8	55523.8	55590.8	55310.9	65468
**5**	52063.5	52231.5	52847.1	52931.1	52580.1	65451

Panel B. Fit indices for models by gender						
**Number of classes**	G-squared	AIC	BIC	CAIC	aBIC	*df*
**1**	83029.2	83093.2	83327.7	83359.7	83226.1	131039
**2**	69588.4	69720.4	70204.1	70270.1	69994.3	131005
**3**	59833.8	60033.8	60766.6	60866.6	60448.8	130971
**4**	55354.2	55622.2	56604.2	56738.2	56178.4	130937
**5**	52507.9	52843.9	54075.1	54243.1	53541.2	130903

This table shows the degrees of freedom , G2 test statistic and information criteria/fit indices used to assess the adequacy of the final model. Examination of the model solutions indicated a model with three clusters had the best fit. The selected model was (1) identified (2) showed parsimony; (3) had sufficient sample sizes in each identified cluster; and (4) the parameter estimates presented a solution that could be both logically and substantively interpreted.

Figure S1. Item-response probabilities for three-class model. Probability of reporting certain health behaviours given latent class among men

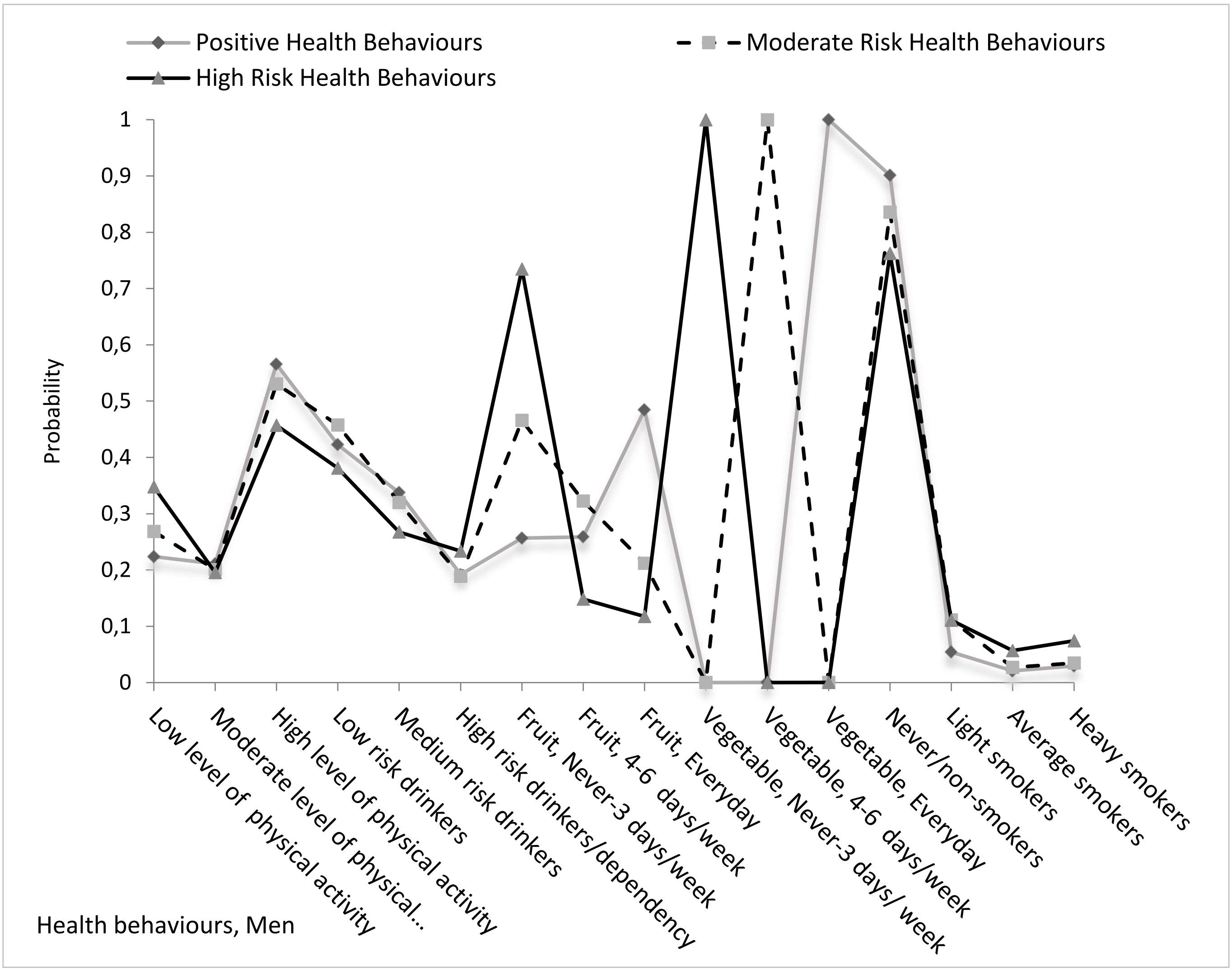

Figure S2. Item-response probabilities for three-cluster model. Probability of reporting certain health behaviours given latent cluster among women.

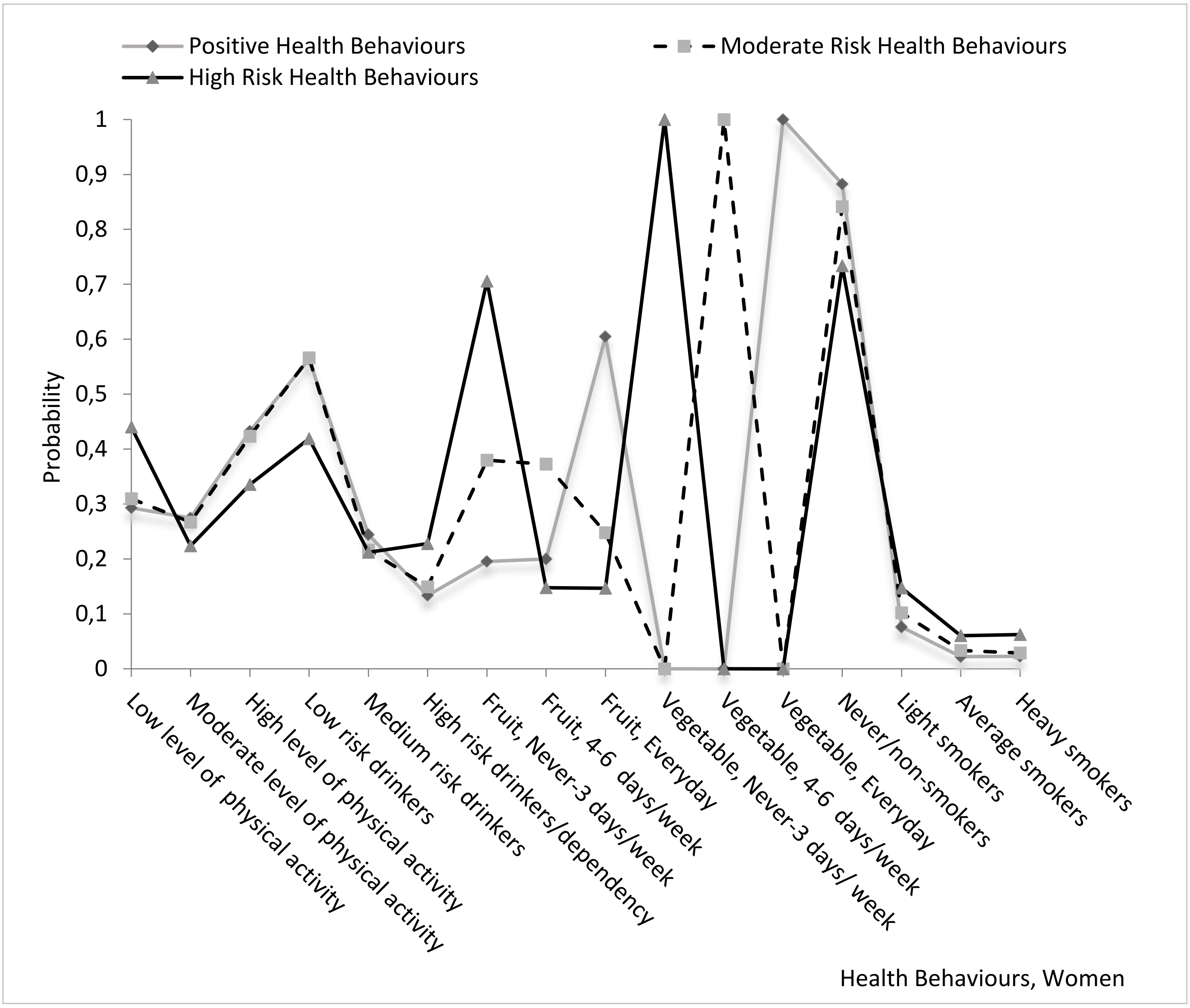

Supplementary Table 3. Marginal changes in mental health problems during the COVID-19 pandemic across the three identified health behaviour clusters.

Panel A. Total Analytical Sample

	Total Sample			Positive Health Behaviours			Moderate risk health behaviours			High Risk Health behaviours		
	Margins	[95% Conf. Interval]		Margins	[95% Conf. Interval]		Margins	[95% Conf. Interval]		Margins	[95% Conf. Interval]	
2017/2019	33,3	31,8	34,8	29,7	27,6	31,8	32,0	29,3	34,7	37,3	34,6	40,0
4/2020	36,7	36,3	37,1	35,4	34,8	36,0	36,9	36,0	37,8	37,7	36,9	38,4
5/2020	36,7	36,3	37,2	35,1	34,5	35,7	37,6	36,6	38,6	37,6	36,8	38,4
6/2020	36,6	36,2	37,1	35,5	34,9	36,1	36,5	35,6	37,4	37,7	36,8	38,6
7/2020	34,9	34,4	35,3	33,4	32,9	34,0	34,5	33,6	35,4	36,3	35,4	37,2
9/2020	34,8	34,3	35,3	33,8	33,1	34,6	35,0	33,9	36,0	35,4	34,5	36,4
11/2020	37,1	36,5	37,8	36,0	35,3	36,8	36,6	35,4	37,9	38,3	37,2	39,5
1/2021	37,4	36,7	38,2	37,2	36,2	38,2	37,4	36,0	38,8	37,6	36,1	39,0

Panel B. Women

	ALL Women			Positive Health Behaviours			Moderate risk health behaviours			High Risk Health behaviours		
	Margins	[95% Conf. Interval]		Margins	[95% Conf. Interval]		Margins	[95% Conf. Interval]		Margins	[95% Conf. Interval]	
2017/2019	35.7	33.8	37.6	31.1	28.9	33.4	36.1	32.9	39.4	40.4	36.6	44.2
4/2020	39.7	39.2	40.2	37.8	37.0	38.5	40.9	39.8	41.9	41.1	40.1	42.1
5/2020	38.4	37.8	38.9	36.5	35.7	37.2	39.6	38.7	40.5	39.7	38.5	40.9
6/2020	38.4	37.8	39.1	37.0	36.1	37.9	38.6	37.3	40.0	40.0	38.7	41.2
7/2020	36.1	35.5	36.6	34.3	33.6	34.9	36.9	35.8	37.9	37.6	36.5	38.6
9/2020	36.2	35.6	36.9	34.6	33.8	35.4	36.9	35.5	38.2	37.6	36.4	38.8
11/2020	38.8	37.9	39.7	36.9	36.1	37.8	39.5	37.5	41.5	40.5	38.7	42.3
1/2021	38.9	37.9	39.8	37.3	36.2	38.4	40.6	38.7	42.4	39.5	37.5	41.4

Panel C. Men

	ALL Men			Positive Health Behaviours			Moderate risk health behaviours			High Risk Health behaviours		
	Margins	[95% Conf. Interval]		Margins	[95% Conf. Interval]		Margins	[95% Conf. Interval]		Margins	[95% Conf. Interval]	
2017/2019	30.4	28.1	32.8	27.3	23.7	31.0	27.5	23.3	31.7	34.4	30.6	38.2
4/2020	33.3	32.6	34.0	31.6	30.6	32.6	32.7	31.3	34.2	34.7	33.6	35.8
5/2020	34.9	34.2	35.7	32.8	31.9	33.7	35.6	33.9	37.2	35.8	34.7	36.9
6/2020	34.6	34.0	35.3	33.1	32.2	33.9	34.3	33.1	35.4	35.7	34.5	37.0
7/2020	33.6	32.7	34.4	32.1	31.2	33.0	32.1	30.6	33.6	35.3	33.8	36.7
9/2020	33.2	32.4	34.1	32.5	31.2	33.9	33.1	31.6	34.6	33.6	32.2	35.0
11/2020	35.3	34.5	36.2	34.6	33.2	35.9	33.8	32.3	35.3	36.5	35.1	38.0
1/2021	35.9	34.7	37.2	37.0	35.2	38.7	34.3	32.1	36.4	36.0	33.9	38.0

Source: Authors’ analysis Understanding Society mainstage survey in 2017/2019 and Understanding Society: COVID-19 Study, waves 1-7
